# Analysis of the contribution of cellular and viral RNA to the packaging of APOBEC3G into HIV-1 virions

**DOI:** 10.1186/1742-4690-4-48

**Published:** 2007-07-16

**Authors:** Mohammad A Khan, Ritu Goila-Gaur, Sandrine Opi, Eri Miyagi, Hiroaki Takeuchi, Sandra Kao, Klaus Strebel

**Affiliations:** 1 Laboratory of Molecular Microbiology, Viral Biochemistry Section, National Institute of Allergy and Infectious Diseases, National Institutes of Health, Building 4, Room 310, 4 Center Drive, MSC 0460, Bethesda, MD 20892-0460, USA

## Abstract

**Background:**

Efficient incorporation of the cellular cytidine deaminase APOBEC3G (APO3G) into HIV-1 virions is necessary for its antiviral activity. Even though cellular RNAs are known to be non-specifically incorporated into virus particles, we have previously found that encapsidation of APO3G into HIV-1 virions is specifically enhanced by viral genomic RNA. Intracellularly, APO3G was found to form large RNA-protein complexes involving a variety of cellular RNAs. The goal of this study was to investigate the possible contribution of host RNAs recently identified in intracellular APO3G ribonucleoprotein complexes to APO3G's encapsidation into HIV-1 virions.

**Results:**

Our results show that 7SL RNA, a component of signal recognition particles, and hY1, hY3, hY4, hY5 RNAs were present in intracellular APO3G complexes and were packaged into HIV-1 particles lacking viral genomic RNA unlike APO3G, which was not packaged in significant amounts into genomic RNA-deficient particles. These results indicate that packaging of 7SL or hY RNAs is not sufficient for the packaging of APO3G into HIV-1 virions. We also tested the encapsidation of several other cellular RNAs including β-actin, GAPDH, α-tubulin, and small nuclear RNAs and determined their effect on the packaging of APO3G into nascent virions. Again, we were unable to observe any correlation between APO3G encapsidation and the packaging of any of these cellular RNAs.

**Conclusion:**

The results from this study support our previous conclusion that viral genomic RNA is a critical determinant for APO3G incorporation into HIV-1 virions. While most cellular RNAs tested in this study were packaged into viruses or virus-like particles we failed to identify a correlation between APO3G encapsidation and the packaging of these cellular RNAs.

## Background

APOBEC3G (APO3G) is a member of the family of cytidine deaminases that in humans include APOBEC1, APOBEC2, seven APOBEC3 variants designated APOBEC3A through 3H, as well as activation-induced deaminase (AID) [1-4]. The protein has potent antiretroviral properties and is expressed in all major target cells susceptible to HIV-1. A crucial prerequisite for antiretroviral activity is the packaging of APO3G into assembling virions. APO3G is efficiently packaged into *vif*-deficient HIV-1 particles but is largely absent from wild type virions [5-11]. A number of studies have shown that packaging of APO3G into virus-like particles (VLP) is mediated through an interaction with the viral Gag precursor [9,11-17]. *In vitro *studies demonstrated that the APO3G-Gag interaction is sensitive to RNase-treatment suggesting a possible role of RNA in APO3G encapsidation [9,11,14,17]. Consistent with these studies, we previously observed that efficient packaging of APO3G into *vif*-deficient HIV-1 particles required the presence of viral genomic RNA [18]. Furthermore, even though small amounts of APO3G were packaged into particles in the absence of viral genomic RNA, such APO3G was sensitive to detergent treatment of the virus and therefore not stably associated with the viral nucleoprotein complex [18]. HIV-1 virions containing genomic RNA packaged approximately 3 times more APO3G and the APO3G found in such virions was largely detergent resistant, indicative of stable association with the viral nucleoprotein complex [18]. Other studies support the significance of viral genomic RNA for the encapsidation of APO3G into HIV-1 particles [16,19,20].

APO3G is an RNA binding protein [21] and recent studies demonstrated that intracellular APO3G can assemble into high molecular mass (HMM) RNA-protein complexes [19,22,23]. Intracellular HMM complexes of APO3G are thought to lack cytidine-deaminase activity and are unable to restrict retrovirus replication [20,22]. Recent analysis of APO3G complexes identified a variety of cellular RNAs including Alu and hY retroelements as well as mRNAs encoding APO3G, ubiquitin, and protein phosphatase 2A [19,23]. On the other hand, messenger RNA encoding α-tubulin was not identified in APO3G complexes [23]. Similarly, β-actin mRNA was found to be absent from [23] or underrepresented in APO3G complexes [19].

Retroviruses including HIV-1 package small cellular RNAs in addition to two copies of viral genomic RNA [24-32]. It is not clear how cellular RNAs are packaged into virions; however, most cellular RNAs appear to be packaged randomly and independent of genomic RNA [28,32]. Furthermore, the efficiency of encapsidation of most of the cellular RNAs seems to reflect their cellular abundance [28,32,33]. One of the first cellular RNAs identified in murine and avian retroviruses is 7SL RNA [34-39]. 7SL RNA is a critical component of the signal recognition particle and is involved in the recognition of the signal peptide during protein translocation across the endoplasmic reticulum [40]. More recently, 7SL RNA was also identified in HIV-1 virions [28,32]; however, so far no functional significance has been associated with the presence of 7SL RNA in retroviral particles.

The current study aimed at the investigation of the possible involvement of cellular RNAs in the encapsidation of APO3G into HIV-1 virions. We focused on RNAs previously identified in intracellular APO3G complexes (e.g. human Y RNAs [23] or HIV-1 RNA [19]) or previously found in retroviral particles (7SL [27,28,32]; snRNAs (U1-U6) [41]). We also analyzed mRNAs previously reported to be excluded from intracellular APO3G complexes (α-tubulin and β-actin [19,23]) and we randomly chose glyceraldehyde-3-phosphate dehydrogenase (GAPDH) to study APO3G binding and virus encapsidation of its mRNA. Our results confirmed the presence of hY1 and hY3 RNAs in intracellular APO3G complexes. In addition, we identified 7SL RNA, U6 snRNA, and GAPDH mRNA as novel components of intracellular APO3G complexes. Only small amounts of α-tubulin mRNA were recovered from APO3G immune complexes as reported before [23]; On the other hand, β-actin mRNA was clearly associated with APO3G complexes in our analysis thus contrasting earlier reports. Most of these RNAs were also packaged into HIV-1 virions. Interestingly, packaging of hY RNAs appeared to be inhibited by the presence of genomic RNA while packaging of other cellular RNAs including 7SL RNA was largely independent of viral genomic RNA. Taken together, our data strongly support a role of viral genomic RNA in the specific encapsidation of APO3G. Our results also demonstrate that cellular RNAs are not sufficient for the encapsidation of APO3G into HIV-1 particles and for the functional association with viral nucleoprotein complexes.

## Results

### Association of APO3G with cellular RNAs

**Figure 1:**
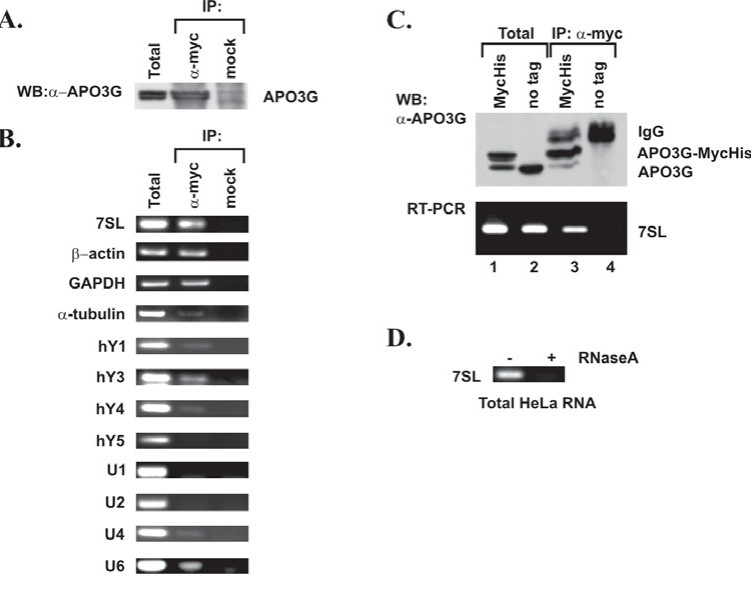
Intracellular association of APO3G and host RNAs. **(A) **Expression and immunoprecipitation of APO3G. HeLa cells (5 × 10^6^) were transfected with 5 μg of pcDNA-Apo3G-MycHis plasmid DNA. Cells were harvested 24 h post transfection. An aliquot of the transfected cells was used for the analysis of APO3G expression as follows: Cell lysates were immunoprecipitated with a polyclonal antibody to the myc epitope tag (α-myc) or were mock immunoprecipitated (mock). Immunoprecipitated samples and total cell lysate (Total) were analyzed for the presence of APO3G by immunoblotting using an APO3G-specific polyclonal peptide antibody. **(B) **The remaining cells from above were used for RT-PCR analysis as follows: Total cellular RNA (Total) or RNA present in the immune complexes (α-myc and mock, respectively) was extracted and used for RT-PCR analysis as described in Methods. Primer pairs were selected for the specific amplification of the RNAs as indicated on the left. Primer sequences are listed in table 1. All RT-PCR reactions were performed simultaneously to minimize experimental error. RT-PCR products were analyzed on 1% agarose gels and visualized by staining with ethidium bromide. **(C) **HeLa cells (5 × 10^6^) were transfected with 5 μg of pcDNA-Apo3G-MycHis plasmid DNA (lanes 1 & 3) or 5 μg of pcDNA-Apo3G (lanes 2 & 4). Cells were harvested 24 h post transfection and analyzed as in panels A and B. **(D) **The specificity of the RT-PCR reaction was validated using 7SL RNA as a substrate. Total cellular RNA from panel B was either left untreated (-) or treated with RNase A (50 μg/ml) for 60 min at 37°C (+) prior to RT-PCR.

Cellular APO3G is present in HMM ribonucleoprotein complexes. Analysis of the RNAs in these complexes revealed the presence of Alu RNAs and small Y RNAs, two of the most prominent non-autonomous mobile genetic elements in human cells [23,42]. We wanted to confirm and extend these observations by further investigating the association of APO3G with other small cellular RNAs such as 7SL RNA, Y RNAs, and U RNAs. Messenger RNAs encoding β-actin, GAPDH, or α-tubulin were included as additional controls for the specificity of APO3G-RNA interactions. HeLa cells were transfected with pcDNA-Apo3G-MycHis DNA. Cells were harvested 24 h after transfection, washed with PBS and divided into two fractions: 30% of the transfected cells were used to isolate total cellular RNA as described in Methods; the remaining 70% of the cells were lysed in Triton X-100 lysis buffer. A sample of the lysate (10%) was used as total protein control for the subsequent immunoblot analysis (Fig. 1A, Total). Equal fractions of the remaining lysate (45% of total lysate each) were either immunoprecipitated with a myc-specific polyclonal antibody (Fig. 1A/B, α-myc) or were exposed to Protein-A beads without antibody (Fig. 1A/B, mock). Half of the immunoprecipitated samples were used for immunoblotting to identify APO3G protein (Fig. 1A). Immunoblot analysis revealed the presence of APO3G in the cell extract (Fig. 1A, Total) and the APO3G immune complex (Fig. 1A, α-myc). As expected, APO3G was absent in the mock immunoprecipitated sample (Fig. 1A, mock). The second half of the precipitated samples was used for RNA extraction. RT-PCR was performed on total RNA and RNA from the immune complexes as described in Methods using a series of primer sets as listed in table 1. All RT-PCR reactions were done simultaneously. RT-PCR of total RNA identified all RNAs in the total cellular extract (Fig. 1B, Total). None of the RNAs was amplified from the mock precipitated sample demonstrating the lack of non-specific binding of these RNAs to Protein A beads (Fig. 1B, mock). In contrast, several of the RNAs, including 7SL, β-actin, and GAPDH, as well as hY3 and U6 RNA were recovered from APO3G immune complexes (Fig. 1B, α-myc). Alpha-tubulin mRNA, as well as hY1, hY4, and U4 RNAs were amplified only inefficiently from the APO3G immune complexes suggesting weak interaction of these RNAs with APO3G (Fig. 1B, α-myc). In contrast, hY5 cytoplasmic RNAs and U1 and U2 small nuclear RNAs did not appear to associate with APO3G immune complexes (Fig. 1B, α-myc).

**Table 1:** Primer sets for RT PCR

**Target**	**Primer Sets**	
	
	**forward**	**reverse**
**α-tubulin^1)^**	cacccgtcttcagggcttcttggttt	catttcaccatctggttggctggctc
**GAPDH^2)^**	gaaggtgaaggtcggagtc	gaagatggtgatgggatttc
**β-actin^3)^**	atggatgatgatatcgccgcg	ctagaagcatttgcggtggacg
**7SL^3)^**	gggctgtagtgcgctatgc	cccgggaggtcaccatatt
**Vif**	gatggcaggtgatgattgtgtgg	ctgtccattcattgtatggc
**hY1^4)^**	ggctggtccgaaggtagtga	aaagactagtcaagtgcagtagtgag
**hY3^4)^**	ggctggtccgagtgcagtg	aaaggctagtcaagtgaagcagtgg
**hY4^4)^**	ggctggtccgagtgcagtg	aaagccagtcaaatttagcagtggg
**hY5^4)^**	agttggtccgagtgttgtggg	aaaacatgcaagctagtcaagcgcg
**U1^5)^**	cctggcaggggagataccatgatcacg	ggggaaagcgcgaacgcagtccccc
**U2^5)^**	cttcttggccttttagctaagatc	ggtgcactgttcctggaggtactgc
**U4^5)^**	gctttgcgcagtggcagtatcg	cagtctccgtagagactgtcaaaaattg
**U6^5)^**	gtgctcgcttcggcagcacatatac	ggaacgcttcacgaatttgcg

1) Eppendorf cMaster RTplusPCR system (Eppendorf Inc. Westbury, NY)

2) Funaki et al. 2003 [54]

3) Onafuwa-Nuga et al. 2006 [28]

4) Chiu et al 2006 [22]

5) Giles et al. 2004 [41]

To rule out non-specific binding of RNAs to the myc-specific antibody in figure 1B, plasmids encoding epitope tagged or untagged APO3G were separately transfected into HeLa cells. Cell extracts were subjected to immunoblot analysis and RT-PCR as described for figure 1B. Myc-tagged and untagged APO3G were efficiently expressed in the transfected cells (Fig. 1C, top panel, lanes 1–2). As expected, untagged APO3G was not immunoprecipitated by the myc-specific antibody (Fig. 1C, top panel, lane 4) while epitope-tagged APO3G-MycHis was identified in the immune complexes (Fig. 1C, top panel, lane 3). A shorter form of APO3G-MycHis co-migrating with the untagged form of APO3G in figure 1C presumably represents C-terminally truncated protein missing part or all of the epitope tag as it was not recognized by epitope-tag-specific antibodies (data not shown). To test non-specific binding of RNA to the myc-specific antibody, we performed RT-PCR as described for figure 1B using 7SL-specific primers. As expected, 7SL RNA was identified in immune complexes of myc-tagged APO3G (Fig. 1C, lower panel, lane 3). However, 7SL RNA was not amplified by RT-PCR from samples containing untagged APO3G (Fig. 1C, lower panel, lane 4). These results demonstrate that the presence of 7SL RNA in immune complexes of myc-tagged APO3G was due to the presence of APO3G and not caused by non-specific binding of the RNA to the myc antibody. Finally, the RT-PCR reaction was sensitive to treatment with RNase A as exemplified by the lack of 7SL RNA amplification in RNase-treated samples (Fig. 1D).

### Cellular RNAs are not sufficient to target APO3G into HIV-1 virions

Previous studies on murine and avian retroviruses found that these viruses encapsidate a variety of host RNAs [24,25,28-31,33,43]. More recent studies have similarly identified cellular RNAs in HIV-1 particles [28,32]. The experiments described above are both consistent with our previous finding that APO3G has RNA binding properties in vitro [21] and other studies demonstrating association of APO3G with cellular RNAs as well as HIV-1 RNA [19,23]. Furthermore, we and others previously reported that viral genomic RNA enhances the encapsidation of APO3G into HIV-1 virions [16,18]. Contrasting these findings, other reports concluded that Gag is sufficient for the encapsidation of APO3G into VLP [9,11-14,16]. Interestingly, the APO3G-Gag interaction was found to be either RNA independent [13] or to be sensitive to RNase-treatment [14] and several studies concluded that nonspecific RNA was critical for APO3G packaging [9,11]. Thus, the parameters determining APO3G packaging into HIV-1 virions remained unclear and warranted further investigation.

**Figure 2:**
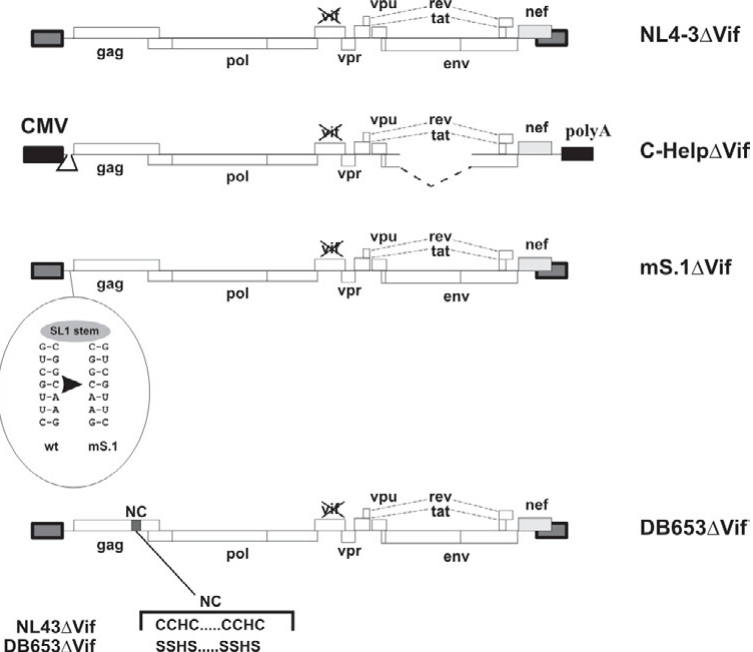
Schematic representation of constructs used in the study. Constructs are discussed in the text. All constructs carry an out-of-frame deletion in the vif gene as described previously [50]. The nucleotide changes in the stem portion of stem-loop 1 region in mS.1ΔVif and the alignment of wild type and DB653 zinc finger residues are shown.

In our next experiment, we compared the packaging of APO3G and cellular RNAs into HIV-1 virions or VLP in an attempt to identify a possible correlation between APO3G packaging and encapsidation of cellular RNAs. Four types of particles were analyzed as shown in Fig. 2. All particles lacked a functional *vif *gene to prevent degradation of APO3G, which would make interpretation of our results more difficult. NL4-3ΔVif served as a positive control; C-HelpΔVif is a helper virus construct lacking both LTRs and carrying deletions in env and in the 5' untranslated region [44]. C-HelpΔVif particles do not package detectable quantities of genomic RNA and we previously found that packaging of APO3G into C-HelpΔVif virions was impaired [18]. The mS.1ΔVif construct carries mutations in stem-loop 1 of the 5' untranslated region of the viral RNA [18] mS.1ΔVif particles contain viral genomic RNA but are impaired in APO3G packaging due to the mutations in the stem loop 1 motif [18]. Finally, DB653ΔVif was included to control for the requirement of NC in RNA and APO3G packaging. DB653ΔVif was derived from DB653 [18,45] and carries SSHS/SSHS mutations in the NC zinc finger motifs. The genomic RNA content of DB653 particles was reported to be less than 10% of wild type virus [45].

**Figure 3:**
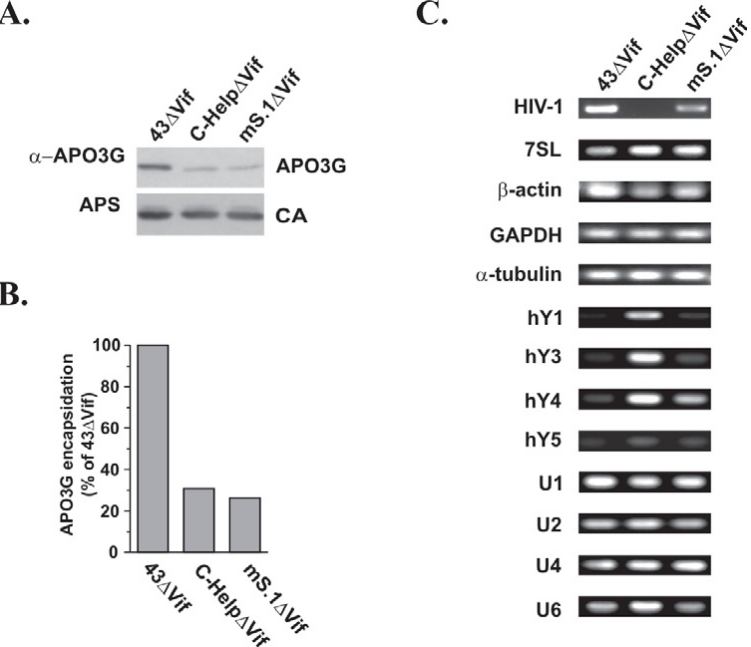
Correlation between cellular and viral RNA encapsidation and APO3G packaging. HeLa cells were co-transfected with pcDNA-APO3G-MycHis together with *vif*-defective variants of either pNL4-3 (43ΔVif), pC-Help (C-HelpΔVif), or mS.1 (mS.1ΔVif). Viruses were harvested 24 h after transfection and purified as described in Methods. **(A) **Virus production and packaging of APO3G was monitored by immunoblot analysis using an aliquot of the purified, concentrated virus preparations. APO3G encapsidation was identified using a polyclonal APO3G-specific peptide antibody. Viral capsid proteins (CA) were identified using an HIV-positive human patient serum (APS). **(B) **APO3G-specific bands in panel A were quantified by densitometric scanning and corrected for fluctuations in capsid levels. Results were calculated relative to APO3G associated with NL4-3ΔVif particles, which was defined as 100%. **(C) **RNAs were extracted from purified, concentrated viruses and amplified by RT-PCR using primer pairs specific for HIV-1 RNA or host RNAs as indicated on the left and detailed in table 1. RT-PCR products were separated on 1% agarose gels and visualized by staining with ethidium bromide.

Particles were produced by transfecting HeLa cells with appropriate plasmid DNAs in the presence of APO3G. Viruses were purified and concentrated as described in Methods. Aliquots were used for immunoblot analysis to determine viral protein content and to verify APO3G packaging (Fig. 3A). Other aliquots of the concentrated viruses were used to extract particle-associated RNA, which was then used for RT-PCR analysis (Fig. 3C). Consistent with our previous report, immunoblot analysis showed that NL4-3ΔVif packaged significantly higher amounts of APO3G than C-HelpΔVif and mS.1ΔVif particles (Fig. 3A). Packaging of APO3G was quantified by densitometric scanning of the APO3G bands. Results were corrected for fluctuations in capsid (CA) levels and are presented as percentage of APO3G packaged into NL4-3ΔVif particles, which was defined as 100% (Fig. 3B). Consistent with our previous data [18], packaging of APO3G into C-HelpΔVif and mS.1ΔVif particles was reduced to about 25–30% of wild type levels.

Equal numbers of particles, as judged by reverse transcriptase activity, were used for extraction of RNA, which was then used for RT-PCR using a series of primers as shown in figure 3C and detailed in table 1. All RT-PCR reactions shown in figure 3C were done simultaneously. Amplification by an HIV-1 specific primer confirmed the presence of genomic RNA in NL4-3ΔVif and mS.1ΔVif particles and verified the lack of detectable amounts of genomic RNA in C-HelpΔVif preparations (Fig. 3C, HIV-1). In contrast, amplification of 7SL RNA as well as β-actin, GAPDH, and α-tubulin mRNAs yielded comparable amounts of PCR products indicative of the presence of similar levels of these cellular RNAs in all three particle preparations. These results suggest that packaging of these RNAs was independent of the presence or absence of viral genomic RNA (Fig. 3C). Similarly, U1, U2, U4, and U6 small nuclear RNAs were amplified with similar efficiency from all three particle preparations. while human Y5 RNA was virtually absent from the particles. On the other hand, hY1, hY3, and hY4 RNAs appeared to be packaged more efficiently into C-HelpΔVif particles than into NL4-3ΔVif and mS.1ΔVif virions. The less efficient packaging of hY1, hY3, and hY4 RNAs into NL4-3ΔVif and mS.1ΔVif particles is unrelated to APO3G encapsidation as APO3G levels in mS.1ΔVif particles were as low as in C-HelpΔVif (Fig. 3A &3B). Importantly, there was no obvious correlation between APO3G packaging and encapsidation of any of the tested cellular RNAs.

### Packaging of hY RNAs requires the NC zinc finger domains

**Figure 4:**
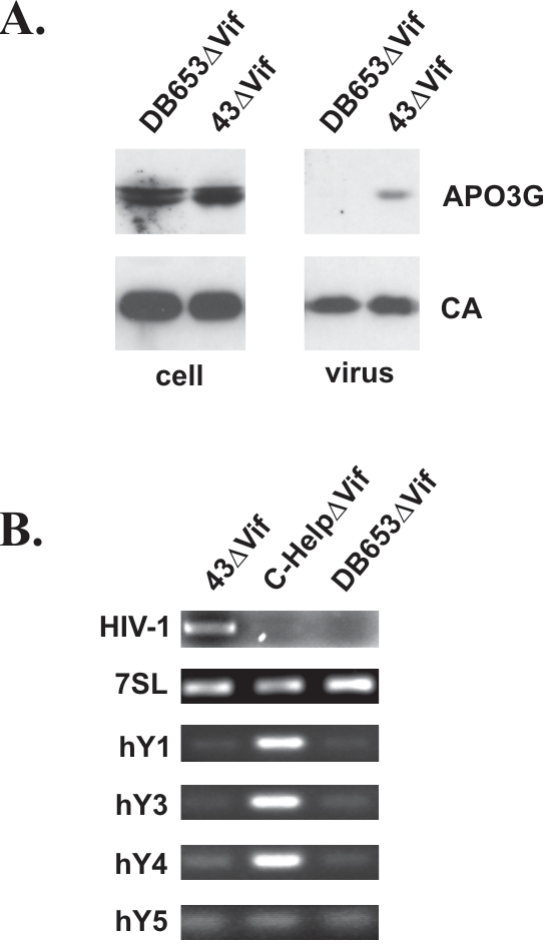
Packaging of hY RNAs requires the NC zinc finger domains. HeLa cells were co-transfected with pcDNA-APO3G-MycHis together with pNL4-3ΔVif (43ΔVif) or pDB653ΔVif. Viruses were harvested 24 h after transfection and purified as described in Methods. **(A) **Virus production and packaging of APO3G was monitored by immunoblot analysis using an aliquot of the purified, concentrated virus preparations. APO3G encapsidation was identified using a polyclonal APO3G-specific peptide antibody. Viral capsid proteins (CA) were identified using an HIV-positive human patient serum (APS). **(B) **RNAs were extracted from purified, concentrated viruses and amplified by RT-PCR using primer pairs specific for HIV-1 RNA or host RNAs as indicated on the left and detailed in table 1. RNA extracted from C-HelpΔVif preparations in figure 3 was included as control. RT-PCR products were separated on 1% agarose gels and visualized by staining with ethidium bromide.

The increased packaging of hY RNAs into particles lacking genomic RNA could indicate a competitive mechanism in which viral genomic RNA competes for a common packaging domain. Since viral genomic RNA is packaged through an interaction with the NC zinc finger domain, we investigated the impact of zinc finger mutations on the packaging of hY RNAs. In addition, we assessed the impact of zinc finger mutations on the packaging of genomic RNA and 7SL RNA as well as APO3G (Fig. 4). NL4-3ΔVif and DB653ΔVif particles were produced from transfected HeLa cells as described for figure 3. Cell lysates and concentrated cell-free virions were subjected to immunoblot analysis to verify comparable amounts of viral Gag proteins and to assess the encapsidation of APO3G into NC zinc finger mutant particles. Consistent with previous reports [9,11-14,16] we found that mutation of the NC zinc finger domain abolished packaging of APO3G into virus-like particles (Fig. 4A, DB653ΔVif).

For RT-PCR analysis, C-HelpΔVif RNA from figure 3C was included for comparison. As before particles were normalized for equal reverse transcriptase activity. RT-PCR analysis using HIV-1-specific primers confirmed the absence of viral genomic RNA in C-HelpΔVif and the DB653ΔVif zinc finger mutant (Fig. 4B). As before, hY5 RNA was virtually absent from all particle preparations including the zinc finger mutant. Interestingly, packaging of 7SL RNA was not affected by mutation of the NC zinc fingers suggesting that 7SL RNA is packaged in an NC-independent manner. In contrast, packaging of hY1, hY3, and hY4 RNAs was critically dependent on intact NC zinc finger domains (Fig. 4B). Thus, packaging of hY RNAs is indeed NC-dependent and the absence of hY RNAs from NL4-3ΔVif particles is best explained by competitive binding of viral genomic RNA and hY RNA to NC.

### 7SL RNA does not promote SRP54 encapsidation

7SL RNA (also referred to as SRP RNA) is a component of the signal recognition particle (SRP), which is critical for the targeting of nascent secretory and membrane proteins to the endoplasmic reticulum membrane (for review see [46]). SRP54 is one of six protein subunits that constitute mammalian SRPs and is responsible for high affinity assembly of 7SL RNA into the SRP complex (reviewed in [47]). Given the fact that 7SL RNA was efficiently packaged into HIV-1 virions, we wanted to test whether intracellular high affinity 7SL RNA-SRP54 interactions would result in the recruitment of SRP54 rather than APO3G into HIV-1 virions.

**Figure 5:**
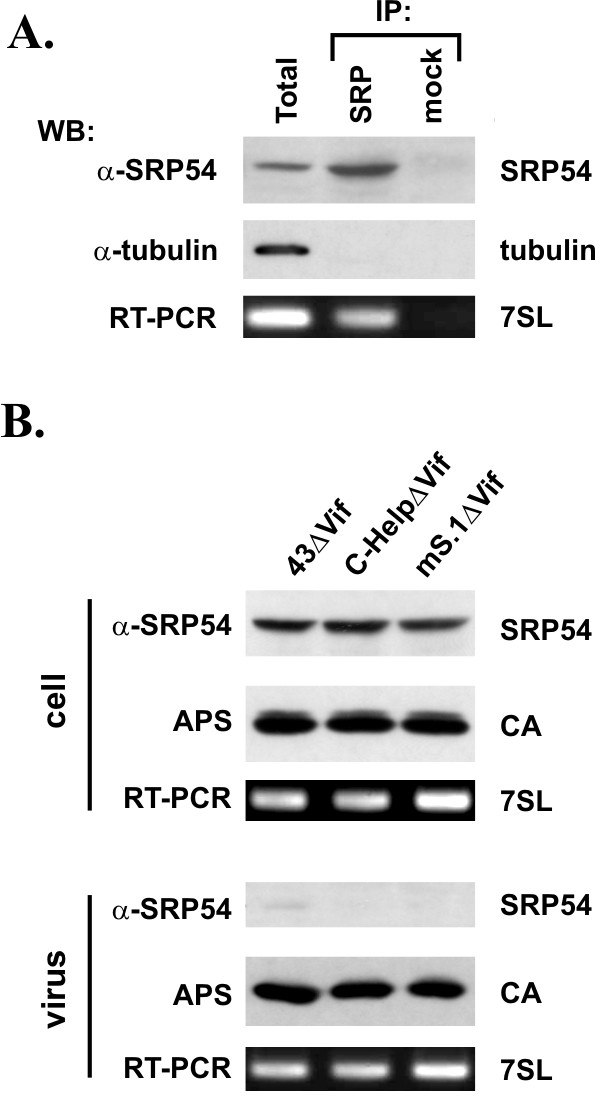
7SL RNA interaction is insufficient for incorporation of SRP54 protein into HIV-1 particles. **(A) **Cell lysates of untransfected HeLa cells were immunoprecipitated with an SRP54-specific antibody (IP) or were mock-precipitated (Ctrl). Aliquots of total cell lysate (Total) and immunoprecipitates were subjected to immunoblot analysis using antibodies to SRP54 (α-SRP54), α-tubulin (α-tubulin). RNA was extracted from remaining cell lysate and immunoprecipitates and used for RT-PCR amplification of 7SL RNA. **(B) **HeLa cells were transfected *vif*-defective variants of either pNL4-3 (43ΔVif), pC-Help (C-HelpΔVif, or mS.1 (mS.1ΔVif). Transfected cells and virus-containing supernatants were harvested 24 h after transfection. Virus-containing supernatants were purified and concentrated as described in Methods. Cell and viral lysates were analyzed by immunoblotting for virus production using an HIV-positive patient serum (APS). Expression and packaging of SRP54 was analyzed using an SRP54-specific antibody oα-SRP54). Total cellular RNA and RNA extracted from concentrated viruses was used for RT-PCR amplification of 7SL RNA.

First, we verified the association of 7SL RNA with SRP54 in normal HeLa cells. For that purpose, HeLa cell lysates were adsorbed to SRP54 reactive autoantibodies and immunoprecipitation of SRP54 was confirmed by immunoblotting using an SRP54-specific antibody (Fig. 5A, top panel, SRP). The specificity of the reaction was verified by the absence of SRP54 protein in mock-immunoprecipitated samples (Fig. 5A, mock) and by the absence of α-tubulin in SRP54-specific and mock precipitates (Fig. 5A, middle panel). Total RNA extracted from the immunoprecipitates revealed the presence of 7SL RNA in SRP54-specific but not in mock immunoprecipitated samples (Fig. 5A, lower panel).

Next, the packaging of SRP54 protein into HIV-1 virions was tested. Virus particles were produced as described for figure 3 except that APO3G was omitted in these samples. Cell lysates and concentrated virus preparations were used for immunoblotting and for RT-PCR analysis as described for figure 3. The results are shown in figure 5B. All cell lysates contained equal amounts of SRP54 and viral capsid proteins as well as 7SL RNA (Fig. 5B, cell). Furthermore, all samples produced comparable amounts of cell-free virions as judged from the immunoblot (Fig. 5B, CA) and packaged comparable amounts of 7SL RNA (Fig. 5B, 7SL). Of note, SRP54 was virtually absent from the virus preparations (Fig. 5B, SRP54), thus confirming and extending a recent study that also did not find SRP54 protein in HIV-1 virions [28]. These results demonstrate that intracellular RNA-protein interactions are not a predictor for subsequent targeting of the proteins into viral particles.

## Discussion

There is general agreement in the literature that APO3G can severely impair replication of HIV-1 and other primate lentiviruses lacking functional Vif proteins. It is also uncontested that the antiviral activity of APO3G – with the notable exception of resting CD4+ T cells [22] – requires the encapsidation of APO3G into nascent virions (for review see [48,49]). However, the mechanism of APO3G encapsidation is not fully understood. *In vitro *studies demonstrated the ability of APO3G to interact with viral Gag protein and the nucleocapsid region of the viral Gag precursor was identified as the likely APO3G binding site [9,12-14,16]. Consistent with this model, studies on virus-like particles demonstrated efficient packaging of APO3G in the absence of viral genomic RNA [9,11-14,16] although some of these studies proposed that non-specific cellular RNA may contribute to APO3G encapsidation [9,11,14,16]. Our own data confirm the importance of NC for encapsidation as APO3G was not encapsidated into a zinc finger mutant (Fig. 4). The absence of APO3G from DB653ΔVif particles combined with the presence of low levels of APO3G in C-HelpΔVif virions (Fig. 3) suggests that APO3G/NC interactions – either with or without support from NC-dependent cellular RNAs – are sufficient for low level packaging of APO3G into virus-like particles. However, the presence of genomic RNA invariably increased the efficiency of APO3G packaging (Figs. 3 &4). Importantly, our previous analysis of helper virus-associated APO3G demonstrated that APO3G packaged through genomic RNA-independent mechanism(s) is sensitive to detergent treatment and thus most likely not associated with the viral nucleoprotein complex [18].

The current study was stimulated by recent reports on the presence of cellular 7SL RNA and snRNAs in HIV-1 virions or retroviral particles [28,32,41] as well as the characterization of cellular RNAs associated with intracellular APO3G [19,23]. Our goal was to test the possible contribution of these or other host RNAs towards the packaging of APO3G into HIV-1 particles. Of the four hY RNAs previously identified in APO3G complexes [23], hY3 was clearly identified in APO3G complexes while hY1 and hY4 only weakly interacted with APO3G in our analysis (Fig. 1B). Among the snRNAs tested, only U6 clearly co-purified with APO3G complexes and U4 showed weak interaction. This finding is interesting since U6 snRNA localizes primarily to the nucleus and does not have a known cytoplasmic function. Surprisingly β-actin mRNA, which was previously reported to be absent from APO3G complexes [19,23] as well as GADPH mRNA clearly co-purified with APO3G in our study. In contrast, we confirmed that α-tubulin mRNA only poorly associated with APO3G. The reasons for these discrepancies are not clear and could be due to differences in experimental conditions. Importantly, however, most RNAs tested in our study were packaged into NL4-3ΔVif virions as well as helper virus and mS.1ΔVif particles (Fig. 3C). Interestingly, comparative RT-PCR analysis demonstrated that hY1, hY3, and hY4 RNAs were more efficiently packaged into C-HelpΔVif particles lacking viral genomic RNA than into particles containing viral genomic RNA (Fig. 3C). Subsequent analysis of an NC mutant revealed that these hY RNAs are packaged through an NC-dependent mechanism. Thus, their inefficient packaging into NL4-3ΔVif and mS.1ΔVif particles may be explained by competitive binding of viral genomic RNA to NC.

U6 snRNA was previously identified in RSV particles [41]. Interestingly, however, U1, U2, and U4 snRNA, all of which were identified in our HIV preparations, were either absent from RSV particles or only present in trace amounts [41]. While it is possible that RSV and HIV differ in the packaging of cellular RNAs, it is also possible that the greater sensitivity of the RT-PCR approach used in our study versus the northern blot analysis employed in the RSV analysis contributed to the different findings. Of note, 7SL RNA despite being packaged in molar excess relative to viral genomic RNA [28] did not promote the packaging of SRP54 protein (Fig. 3B) consistent with a recent report [28]. Thus, despite the high affinity interaction of 7SL RNA with SRP54, such intracellular interaction was insufficient to promote packaging of SRP54 into cell-free virions. Similarly, packaging of RNAs previously found in association with intracellular APO3G complexes was insufficient to support APO3G encapsidation. Thus, we did not observe a correlation between the packaging of cellular RNAs into HIV-1 particles and encapsidation of APO3G. The exclusion of APO3G from C-HelpΔVif particles lacking genomic RNA but containing high levels of cellular RNAs and the absence of APO3G from mS.1ΔVif particles containing genomic RNA with mutations in the stem-loop 1 region of the 5' untranslated region point to a role of viral genomic RNA in the packaging of APO3G. We cannot formally rule out that other, thus far unidentified cellular RNA species contribute to the packaging of APO3G into virus particles; however, this seems unlikely since we would have to posit that such RNAs are specifically excluded from C-HelpΔVif and mS.1ΔVif particles.

## Conclusion

We have demonstrated that *vif*-defective HIV-1 particles package a variety of cellular RNAs. Most of the cellular RNAs tested, except hY RNAs, were packaged independent of viral genomic RNA. Packaging of hY RNAs was NC-dependent and inhibited by viral genomic RNA. In all experiments, APO3G packaging correlated well with the presence of viral genomic RNA but not with the presence of any of the cellular RNAs tested. Thus, our data do not support a model in which APO3G is packaged through non-specific or specific interaction with cellular RNAs. In particular, we can rule out that packaging of 7SL RNA is sufficient for the encapsidation of APO3G. Instead, we propose that packaging of APO3G into virus particles is mediated through interaction with viral genomic RNA.

## Methods

### Plasmids

The *vif*-defective molecular clone pNL4-3ΔVif [50] was used for the production of *vif*-defective HIV-1 virus stocks. Plasmid pC-HelpΔVif was used for the production of *vif*-defective Ψ^- ^virus-like particles (VLP). These particles contain undetectable levels of viral genomic RNA [18]. Plasmid pNL4-3mS.1ΔVif carries mutations in stem-loop 1 of the 5'-untranslated region [51] and was constructed by subcloning the mutated stem-loop 1 region into the *vif*-defective pNL4-3 vector [18]. NL4-3mS.1ΔVif particles are Ψ^+ ^but do not support the encapsidation of APO3G [18]. A *vif*-defective variant of DB653 [45] was constructed by transferring the Gag region of DB653 into pNL4-3Vif(-) using standard cloning techniques. The structures of these constructs are schematically shown in figure 2. Construction of pcDNA-Apo3G-MycHis for the expression of C-terminally epitope-tagged wild type human APO3G proteins was described previously [7] and untagged version, pcDNA-Apo3G, was construction by introducing a stop coding at the end of the APO3G gene [52].

### Tissue culture and transfection

HeLa cells, which do not express endogenous APO3G, were propagated in Dulbecco's modified Eagles medium (DMEM) containing 10% fetal bovine serum (FBS). For transfection, HeLa cells were grown in 25 cm^2 ^flasks to about 80% confluency. Cells were transfected using LipofectAMINE PLUS™ (Invitrogen Corp, Carlsbad CA) following the manufacturer's recommendations. A total of 5 μg of plasmid DNA per 25 cm^2 ^flask (5 × 10^6 ^cells) was generally used. Cells were harvested 24 h post transfection.

### Preparation of virus stocks

Virus stocks were prepared by transfecting HeLa cells with pNL4-3ΔVif, pC-HelpΔVif, or pNL4-3mS.1ΔVif DNAs in the presence or absence of APO3G expression vector as indicated in the text. Virus-containing supernatants were harvested 24 h after transfection. Cellular debris was removed by centrifugation (5 min, 1500 rpm) and clarified supernatants were filtered (0.45 μm) to remove residual cellular contaminants. For immunoblot analysis of viral proteins and RNA extraction, virus-containing supernatants (7 ml) were concentrated by ultracentrifugation through 2 ml of 20% sucrose in PBS as described previously [7].

### Antisera

APO3G was identified using a polyclonal rabbit serum against a synthetic peptide comprising the 17 C-terminal residues of APO3G. Serum from an HIV-positive patient (APS) was used to detect HIV-1-specific capsid (CA) proteins. Tubulin was identified using an α-tubulin-specific monoclonal antibody (Sigma-Aldrich, Inc., St. Louis MO). SRP54 protein was detected with a SRP54-specific monoclonal antibody (BD Biosciences, San Jose, CA). Immunoprecipitation of APO3G was done using a polyclonal antibody raised against the myc tag (Sigma-Aldrich, Inc., St. Louis, MO). A human SRP54-reactive autoimmune serum was used for immunoprecipitation of SRP54 protein (kind gift of Frederick W. Miller, NIEHS, NIH, Bethesda, MD, USA).

### Immunoblotting

HeLa cells transfected with APO3G were used to detect cellular APO3G expression and untransfected HeLa cells were used for the detection of endogenous SRP54 protein by immunoblotting with appropriate antibodies. For immunoblot analysis of cellular proteins, whole cell lysates were prepared as follows. Cells were washed once with PBS, suspended in 450 μl/10^7 ^cells with X-100 lysis buffer (50 mM Tris-HCL pH7.5, 150 mM NaCl, 0.5% Triton X-100). For Western blot analysis 50 μl aliquot was taken and mixed with equal volume of sample buffer (4% sodium dodecyl sulfate [SDS], 25 mM Tris-HCL, pH 6.8, 10% 2-mercaptoethanol, 10% glycerol, and 0.002% bromphenol blue). Proteins were solubilized by boiling for 5 min at 95°C with occasional vortexing of the samples to shear chromosomal DNA. Residual insoluble material was removed by centrifugation (2 min, 15,000 rpm in an Eppendorf Minifuge). For immunoblot analysis of virus-associated proteins, concentrated viral pellets were suspended in a 1:1 mixture of PBS and sample buffer and boiled. Cell lysates and viral extracts were subjected to SDS-polyacrylamide gel electrophoresis; proteins were transferred to polyvinylidene difluoride membranes and reacted with appropriate antibodies as described in the text. Membranes were then incubated with horseradish peroxidase-conjugated secondary antibodies (Amersham Bioscience, Piscataway, NJ) and visualized by enhanced chemiluminescence (Amersham Bioscience).

### Immunoprecipitation analysis

HeLa cells were transfected with pcDNA-APO3G-MycHis. Cells were harvested at 24 h post transfection cell lysates were prepared as follows: Cells were divided into two unequal fractions (30% and 70%). The larger fraction was used for immunoprecipitation studies and the smaller fraction was used for RNA extraction (see below). For immunoprecipitation, cells were washed once with PBS and lysed in 450 μl of lysis buffer (50 mM Tris, pH7.5, 150 mM, NaCl 0.5% and Triton X-100). The cell extracts were clarified by centrifugation (13,000 × g, 3 min) and the supernatant was incubated on a rotating wheel for 1 h at 4°C with protein A-Sepharose beads (Sigma-Aldrich, Inc., St. Louis MO) coupled with (IP) or without (Ctrl) anti-myc rabbit polyclonal antibody (Sigma-Aldrich, Inc., St. Louis MO). Immunocomplexes were washed three times with wash buffer (50 mM Tris, 300 mM NaCl, and 0.1% Triton X-100 (pH 7.4). Bound proteins were eluted form beads by heating in sample buffer for 5 min at 95°C and analyzed by immunoblotting using antibodies as indicated in the text. For immunoprecipitation of APO3G-RNA complexes, cell extracts were subjected to immunoprecipitation by antibody covered beads or control beads as described above and washed three times with RNA-protein binding buffer (20 mM HEPES, 25 mM KCl, 7 mM 2-Mercaptoethanol, 5% Glycerol and 0.1% NP-40). Bound RNA was then extracted as described below.

### RNA extraction

Total cellular RNA was extracted from untransfected and transfected HeLa cells using the RNeasy RNA extraction kit (QIAGEN, Valencia, CA) following the manufacturer's instructions. To isolate RNA from immunocomplexes, beads were washed three times with RNA-protein binding buffer (20 mM HEPES, 25 mM KCl, 7 mM 2-Mercaptoethanol, 5% Glycerol and 0.1% NP-40). RNA was then extracted using RNeasy RNA extraction kit. For isolation of SRP54-associated RNA, SRP54 was precipitated with SRP54-reactive human autoantibodies derived from a patient with polymyositis ([53]; gift of Frederick W Miller, NIEHS, NIH, Bethesda, MD, USA). RNA was then extracted from the immunocomplexes as before

### RT-PCR

RNA extracted from cells, viruses, or immunocomplexes was treated with RNase-free DNase I (10 units, 30 min, 37°C) prior to the RT-PCR reaction. RNA concentrations were determined by spectrophotometry. RT-PCR was performed using equal amounts of RNA and the one-step RT-PCR kit (QIAGEN, Valencia, CA) according to the manufacturer's instruction. Primers for the amplification of specific RNAs are listed in table 1. RNA was first reverse transcribed at 50°C for 30 minutes followed by 30 PCR cycles (denaturation at 94°C; 15 sec; annealing at 55°C, 30 sec; and extension at 72°C, 1 min) and one 10-minute extension cycle at 72°C. RT-PCR products were mixed with DNA loading buffer (EDTA 20 mM, TAE 5×, Glycerol 50% and 0.002% Bromphenol Blue dye), electrophoresed in 1% agarose gels, and visualized by staining with ethidium bromide. A DNA size marker was run in parallel.

## Competing interests

The author(s) declare that they have no competing interests.

## Authors' contributions

M.K. conceived the study, was leading the execution of the experiments, and participated in the writing of the manuscript. K.S. coordinated and supervised the study and was involved in the writing of the manuscript. R.G., S.O., E.M., H.T., and S.K. participated in virus production and sample preparation and provided critical comments on the manuscript. All authors read and approved the final manuscript.

## References

[B1] Conticello SG, Thomas CJ, Petersen-Mahrt SK, Neuberger MS (2005). Evolution of the AID/APOBEC family of polynucleotide (deoxy)cytidine deaminases. Mol Biol Evol.

[B2] Jarmuz A, Chester A, Bayliss J, Gisbourne J, Dunham I, Scott J, Navaratnam N (2002). An anthropoid-specific locus of orphan C to U RNA-editing enzymes on chromosome 22. Genomics.

[B3] Rogozin IB, Basu MK, Jordan IK, Pavlov YI, Koonin EV (2005). APOBEC4, a New Member of the AID/APOBEC Family of Polynucleotide (Deoxy)cytidine Deaminases Predicted by Computational Analysis. Cell Cycle.

[B4] Wedekind JE, Dance GS, Sowden MP, Smith HC (2003). Messenger RNA editing in mammals: new members of the APOBEC family seeking roles in the family business. Trends Genet.

[B5] Mariani R, Chen D, Schrofelbauer B, Navarro F, Konig R, Bollman B, Munk C, Nymark-McMahon H, Landau NR (2003). Species-specific exclusion of APOBEC3G from HIV-1 v irions by Vif. Cell.

[B6] Marin M, Rose KM, Kozak SL, Kabat D (2003). HIV-1 Vif protein binds the editing enzyme APOBEC3G and induces its degradation. Nat Med.

[B7] Kao S, Khan MA, Miyagi E, Plishka R, Buckler-White A, Strebel K (2003). The human immunodeficiency virus type 1 Vif p rotein reduces intracellular expression and inhibits packaging of APOBEC3G (CEM15), a cellular inhibitor of virus infectivity. J Virol.

[B8] Yu X, Yu Y, Liu B, Luo K, Kong W, Mao P, Yu XF (2003). Induction of APOBEC3G ubiquitination and degradation by an HIV-1 Vif-Cul5-SCF complex. Science.

[B9] Zennou V, Perez-Caballero D, Gottlinger H, Bieniasz PD (2004). APOBEC3G incorporation into human immunodeficiency virus type 1 particles. J Virol.

[B10] Mangeat B, Turelli P, Caron G, Friedli M, Perrin L, Trono D (2003). Broad antiretroviral defence by human APOBEC3G through lethal editing of nascent reverse transcripts. Nature.

[B11] Svarovskaia ES, Xu H, Mbisa JL, Barr R, Gorelick RJ, Ono A, Freed EO, Hu WS, Pathak VK (2004). Human apolipoprotein B mRNA-editing enzyme-catalytic polypeptide-like 3G (APOBEC3G) is incorporated into HIV-1 virions through interactions with viral and nonviral RNAs. J Biol Chem.

[B12] Alce TM, Popik W (2004). APOBEC3G is incorporated into virus-like particles by a direct interaction with HIV-1 Gag nucleocapsid protein. J Biol Chem.

[B13] Cen S, Guo F, Niu M, Saadatmand J, Deflassieux J, Kleiman L (2004). The interaction between HIV-1 Gag and APOBEC3G. J Biol Chem.

[B14] Schafer A, Bogerd HP, Cullen BR (2004). Specific packaging of APOBEC3G into HIV-1 virions is mediated by the nucleocapsid domain of the gag polyprotein precursor. Virology.

[B15] Douaisi M, Dussart S, Courcoul M, Bessou G, Vigne R, Decroly E (2004). HIV-1 and MLV Gag proteins are sufficient to recruit APOBEC3G into virus-like particles. Biochem Biophys Res Commun.

[B16] Luo K, Liu B, Xiao Z, Yu Y, Yu X, Gorelick R, Yu XF (2004). Amino-terminal region of the human immunodeficiency virus type 1 nucleocapsid is required for human APOBEC3G packaging. J Virol.

[B17] Burnett A, Spearman P (2007). APOBEC3G Multimers Are Recruited to the Plasma Membrane for Packaging into Human Immunodeficiency Virus Type 1 Virus-Like Particles in an RNA-Dependent Process Requiring the NC Basic Linker. J Virol.

[B18] Khan MA, Kao S, Miyagi E, Takeuchi H, Goila-Gaur R, Opi S, Gipson CL, Parslow TG, Ly H, Strebel K (2005). Viral RNA is required for the association of APOBEC3G with human immunodeficiency virus type 1 nucleoprotein complexes. J Virol.

[B19] Kozak SL, Marin M, Rose KM, Bystrom C, Kabat D (2006). The anti-HIV-1 editing enzyme APOBEC3G binds HIV-1 RNA and messenger RNAs that shuttle between polysomes and stress granules. J Biol Chem.

[B20] Soros VB, Yonemoto W, Greene WC (2007). Newly synthesized APOBEC3G is incorporated into HIV virions, inhibited by HIV RNA, and subsequently activated by RNase H. PLoS Pathog.

[B21] Iwatani Y, Takeuchi H, Strebel K, Levin JG (2006). Biochemical Activities of Highly Purified, Catalytically Active Human APOBEC3G: Correlation with Antiviral Effect. J Virol.

[B22] Chiu YL, Soros VB, Kreisberg JF, Stopak K, Yonemoto W, Greene WC (2005). Cellular APOBEC3G restricts HIV-1 infection in resting CD4+ T cells. Nature.

[B23] Chiu YL, Witkowska HE, Hall SC, Santiago M, Soros VB, Esnault C, Heidmann T, Greene WC (2006). High-molecular-mass APOBEC3G complexes restrict Alu retrotransposition. Proc Natl Acad Sci USA.

[B24] Adkins B, Hunter T (1981). Identification of a packaged cellular mRNA in virions of rous sarcoma virus. J Virol.

[B25] Aronoff R, Linial M (1991). Specificity of retroviral RNA packaging. J Virol.

[B26] Berkowitz R, Fisher J, Goff SP (1996). RNA packaging. Curr Top Microbiol Immunol.

[B27] Onafuwa-Nuga AA, King SR, Telesnitsky A (2005). Nonrandom packaging of host RNAs in moloney murine leukemia virus. J Virol.

[B28] Onafuwa-Nuga AA, Telesnitsky A, King SR (2006). 7SL RNA, but not the 54-kd signal recognition particle protein, is an abundant component of both infectious HIV-1 and minimal virus-like particles. RNA.

[B29] Muriaux D, Mirro J, Harvin D, Rein A (2001). RNA is a structural element in retrovirus particles. Proc Natl Acad Sci USA.

[B30] Jiang M, Mak J, Ladha A, Cohen E, Klein M, Rovinski B, Kleiman L (1993). Identification of tRNAs incorporated into wild-type and mutant human immunodeficiency virus type 1. J Virol.

[B31] Linial M, Medeiros E, Hayward WS (1978). An avian oncovirus mutant (SE 21Q1b) deficient in genomic RNA: biological and biochemical characterization. Cell.

[B32] Rulli SJ, Hibbert CS, Mirro J, Pederson T, Biswal S, Rein A (2007). Selective and Nonselective Packaging of Cellular RNAs in Retrovirus Particles. J Virol.

[B33] Gallis B, Linial M, Eisenman R (1979). An avian oncovirus mutant deficient in genomic RNA: characterization of the packaged RNA as cellular messenger RNA. Virology.

[B34] Erikson RL (1969). Studies on the RNA from avian myeloblastosis virus. Virology.

[B35] Bishop JM, Levinson WE, Sullivan D, Fanshier L, Quintrell N, Jackson J (1970). The low molecular weight RNAs of Rous sarcoma virus. II. The 7 S RNA. Virology.

[B36] Erikson E, Erikson RL, Henry B, Pace NR (1973). Comparison of oligonucleotides produced by RNase T1 digestion of 7 S RNA from avian and murine oncornaviruses and from uninfected cells. Virology.

[B37] Faras AJ, Garapin AC, Levinson WE, Bishop JM, Goodman HM (1973). Characterization of the low-molecular-weight RNAs associated with the 70S RNA of Rous sarcoma virus. J Virol.

[B38] Sawyer RC, Dahlberg JE (1973). Small RNAs of Rous sarcoma virus: characterization by two-dimensional polyacrylamide gel electrophoresis and fingerprint analysis. J Virol.

[B39] Walker TA, Pace NR, Erikson RL, Erikson E, Behr F (1974). The 7S RNA common to oncornaviruses and normal cells is associated with polyribosomes. Proc Natl Acad Sci USA.

[B40] Walter P, Blobel G (1982). Signal recognition particle contains a 7S RNA essential for protein translocation across the endoplasmic reticulum. Nature.

[B41] Giles KE, Caputi M, Beemon KL (2004). Packaging and reverse transcription of snRNAs by retroviruses may generate pseudogenes. RNA.

[B42] Perreault J, Noel JF, Briere F, Cousineau B, Lucier JF, Perreault JP, Boire G (2005). Retropseudogenes derived from the human Ro/SS-A autoantigen-associated hY RNAs. Nucleic Acids Res.

[B43] Ikawa Y, Ross J, Leder P (1974). An association between globin messenger RNA and 60S RNA derived from Friend leukemia virus. Proc Natl Acad Sci USA.

[B44] Mochizuki H, Schwartz JP, Tanaka K, Brady RO, Reiser J (1998). High-titer human immunodeficiency virus type 1-based vector systems for gene delivery into nondividing cells. J Virol.

[B45] Guo J, Wu T, Anderson J, Kane BF, Johnson DG, Gorelick RJ, Henderson LE, Levin JG (2000). Zinc finger structures in the human immunodeficiency virus type 1 nucleocapsid protein facilitate efficient minus- and plus-strand transfer. J Virol.

[B46] Keenan RJ, Freymann DM, Stroud RM, Walter P (2001). The signal recognition particle. Annu Rev Biochem.

[B47] Doudna JA, Batey RT (2004). Structural insights into the signal recognition particle. Annu Rev Biochem.

[B48] Hache G, Mansky LM, Harris RS (2006). Human APOBEC3 proteins, retrovirus restriction, and HIV drug resistance. AIDS Rev.

[B49] Chiu YL, Greene WC (2006). APOBEC3 Cytidine Deaminases: Distinct Antiviral Actions along the Retroviral Life Cycle. J Biol Chem.

[B50] Karczewski MK, Strebel K (1996). Cytoskeleton association and virion incorporation of the human immunodeficiency virus type 1 Vif protein. J Virol.

[B51] Clever JL, Parslow TG (1997). Mutant human immunodeficiency virus type 1 genomes with defects in RNA dimerization or encapsidation. J Virol.

[B52] Opi S, Takeuchi H, Kao S, Khan MA, Miyagi E, Goila-Gaur R, Iwatani Y, Levin JG, Strebel K (2006). Monomeric APOBEC3G Is Catalytically Active and Has Antiviral Activity. J Virol.

[B53] Romisch K, Miller FW, Dobberstein B, High S (2006). Human autoantibodies against the 54 kDa protein of the signal recognition particle block function at multiple stages. Arthritis Res Ther.

[B54] Funaki H, Nishimura G, Harada S, Ninomiya I, Terada I, Fushida S, Tani T, Fujimura T, Kayahara M, Shimizu K, Ohta T, Miwa K (2003). Expression of vascular endothelial growth factor D is associated with lymph node metastasis in human colorectal carcinoma. Oncology.

